# Highlighting New Perspectives on Musculoskeletal Infections

**DOI:** 10.3390/microorganisms12010226

**Published:** 2024-01-22

**Authors:** Michele Fiore, Andrea Sambri

**Affiliations:** 1 Department of Medical and Surgical Sciences, University of Bologna, 40138 Bologna, Italy; 2 Orthopaedics and Traumatology Unit, IRCCS Azienda Ospedaliero Universitaria di Bologna, 40138 Bologna, Italy

The treatment of musculoskeletal and prosthetic joint infections represents a considerable challenge for patients, healthcare providers, and the healthcare system because of the high number of treatment failures and the significant economic burden [1]. Thus, attempts to unveil new possibilities through research are crucial.

This Special Issue, “Prosthetic and Bone Infections: A Multidisciplinary Approach”, attempted to highlight multidisciplinarity in the field of musculoskeletal infections by gathering data and experiences on the management of patients with bone, soft tissue, joint, periprosthetic, or hardware-related infections. Beyond the strictly clinical aspects, demonstrated in the papers addressing the need for an “orthoplastic” approach on the most demanding cases [2], we were impressed by the evident interest in the laboratory and instrumental aspects. This was evident in the attention paid to establishing novel imaging methods, as well as in the focus on biological and molecular considerations [3]. Genetics, molecular biology, and microbiology are fast-evolving fields in the context of musculoskeletal infections. Deepening the understanding of pathogen biology will undoubtedly activate new processes for the diagnostic optimization and individualization of treatments [4]. Moreover, further attention is simultaneously needed for an in-depth study of the correlations between patient genetics and biology (including metabolic aspects) and infectious outcomes. Another interesting perspective provided in this Special Issue considers the significant contributions that new technologies can provide to surgical treatments, as in the case of bone substitutes and biocompatible materials [5].

A total of 10 papers have been published in this Special Issue, thus highlighting the importance of multidisciplinarity in bone and prosthetic joint infections. The collaboration between orthopedic and plastic surgeons, infectious disease specialists, and microbiologists with experience in molecular biology is necessary.

In conclusion, we firmly believe that in the field of musculoskeletal infections, promoting monothematic but multidisciplinary lines of research (ranging from clinical to biological to engineering aspects) is one of the responsibilities of modern healthcare providers. The interplay and “contamination” of knowledge and expertise can stimulate cross-connections and usher in new research directions. We hope that this Special Issue provides a focused and significant demonstration of this perspective.
